# Glycan Profiling Shows Unvaried *N*-Glycomes in MSC Clones with Distinct Differentiation Potentials

**DOI:** 10.3389/fcell.2016.00052

**Published:** 2016-05-31

**Authors:** Katherine M. Wilson, Jane E. Thomas-Oates, Paul G. Genever, Daniel Ungar

**Affiliations:** ^1^Department of Biology, University of YorkYork, UK; ^2^Department of Chemistry and Centre of Excellence in Mass Spectrometry, University of YorkYork, UK

**Keywords:** MALDI-MS, *N*-glycan, FANGS, immunomodulatory MSCs, multi-lineage differentiation, self-renewal

## Abstract

Different cell types have different *N*-glycomes in mammals. This means that cellular differentiation is accompanied by changes in the *N*-glycan profile. Yet when the *N*-glycomes of cell types with differing fates diverge is unclear. We have investigated the *N*-glycan profiles of two different clonal populations of mesenchymal stromal cells (MSCs). One clone (Y101), when differentiated into osteoblasts, showed a marked shift in the glycan profile toward a higher abundance of complex *N*-glycans and more core fucosylation. Yet chemical inhibition of complex glycan formation during osteogenic differentiation did not prevent the formation of functional osteoblasts. However, the *N*-glycan profile of another MSC clone (Y202), which cannot differentiate into osteoblasts, was not significantly different from that of the clone that can. Interestingly, incubation of Y202 cells in osteogenic medium caused a similar reduction of oligomannose glycan content in this non-differentiating cell line. Our analysis implies that the *N*-glycome changes seen upon differentiation do not have direct functional links to the differentiation process. Thus *N*-glycans may instead be important for self-renewal rather than for cell fate determination.

## Introduction

Mesenchymal stromal cells (MSCs) are a heterogeneous population of cells that contains both adult multipotent and immunomodulatory cell types (Nauta and Fibbe, 2007). They can be isolated from several locations in the human body by exploiting their ability to adhere to plastic. MSCs are often distinguished from other cells types by the expression of CD105, CD73, CD90 cell surface markers and the absence of hematopoietic markers such as CD45, CD43, CD14, and CD19 (Dominici et al., 2006). Once isolated, MSCs can be induced to differentiate into bone, cartilage or fat cells, but not all the cells in a heterogeneous primary isolate behave the same way during a differentiation experiment. In fact, clonal sub-populations of primary MSCs show different features than the parent population. Some clones can differentiate into all three lineages. Others are only capable of dual or single lineage differentiation, while some do not differentiate at all (Pittenger et al., 1999). In addition, MSCs have been observed to provide immunomodulatory functions, a role that is shared by both differentiating and non-differentiating cells (Bartholomew et al., 2002; James et al., 2015). Given the range of phenotypic characteristics and the lack of decisive markers, the precise identification of MSCs is not trivial in a primary mixture. Moreover, the characterization of different MSC lines derived from primary cells would also benefit from the elucidation of further defining molecular features.

We generated single cell-derived clones from immortalized bone-marrow MSCs. Importantly, different clones obtained in the course of immortalization represent the various cell types contained in the heterogeneous primary mixture. Four such clones have been characterized in detail, two of which are capable of differentiation, while the other two represent MSCs with enhanced immunomodulatory features that cannot differentiate (James et al., 2015). The two differentiating lines (Y101 and Y201) are similar in their gene expression profiles, and both show tri-lineage differentiation potential, although Y101 cells are primed for osteogenic differentiation, and do not differentiate efficiently into adipocytes. The non-differentiating lines (Y102 and Y202) exhibit little differentiation capacity with possibly a very weak adipogenic potential, but express a significant proportion of pro-inflammatory genes, with increased IL-7 production. They are characterized by the unique cell-surface marker CD317, and represent approximately 10% of the mixed primary MSC population. Apart from the inflammatory markers, there are as yet no clear differences in the four lines that would indicate why the '01 lines can and the '02 lines cannot differentiate. These MSC lines thus represent excellent models to study the molecular characteristics of the various MSC populations found *in vivo*.

Cellular differentiation is accompanied by a change in glycosylation patterns. For example, the antigens marking different stages of embryonic stem cell development are glycans (Gooi et al., 1981). Along similar lines, the *N*-glycome undergoes remodeling during embryonic cell differentiation (Amano et al., 2010). Glycosylation differences also exist between cells that are at the same stage of differentiation, but have different lineage commitments, such as the extensive differences observed in lectin binding affinities for neuronal and mesenchymal progenitor cells (Dodla et al., 2011). The differentiation of primary MSCs also leads to glycosylation changes. Glycomic comparison of adipocytes and their parental MSCs (a heterogeneous primary population) has uncovered increased sialylation and decreased branching of complex *N*-glycans as a consequence of differentiation (Hamouda et al., 2013). In contrast, osteogenic differentiation of another heterogeneous primary cell population has been associated with a decrease in the levels of oligomannose type *N*-glycans (Heiskanen et al., 2009). Although many other changes were observed between MSCs and the derived osteoblasts, detailed quantification of the differences is skewed by the fact that glycan permethylation was not used prior to MALDI-MS analysis, which prevents the quantification of relative glycan abundances (Wada et al., 2007).

While glycosylation differences are a well-accepted feature of different cell types, the functional significance of these variations is less clear. Moreover, it is unknown whether MSC sub-types with different properties, such as differentiation-competent MSCs vs. primarily immunomodulatory MSCs, have differences in their glycosylation profiles. We therefore asked if differences in the glycosylation state between the various MSC sub-populations in our model cultures could be observed, and could potentially contribute to phenotypic differences between the MSC subtypes within a primary mixture. In accordance with previous findings (Heiskanen et al., 2009), we observed a robust change in the *N*-glycan profile during osteogenesis of the tested homogeneous MSC line. Interestingly, detailed quantitative *N*-glycan profiling showed remarkable similarity for two different immortalized MSC lines (Y101 and Y202) despite their differing phenotypes.

## Materials and methods

### Cell culture and osteogenic differentiation

Cells were cultured in basal medium composed of DMEM (high glucose, pyruvate, no glutamine) supplemented with 10% fetal bovine serum, 1% penicillin/streptomycin, and 1% GlutaMax-I. To stimulate osteogenesis, cells were seeded at a density of 20,000 cells/cm^2^ and 50 μg/mL ascorbic acid, 5 mM β-glycerophosphate, and 10 nM dexamethasone were added to basal medium. Culture medium was changed every 3–4 days during assays. For histochemistry cells were cultured in 24-well dishes, for glycan profiling in 10 cm plates.

### Preparation of *N*-glycans for mass spectrometry

The filter-aided *N*-glycan separation (FANGS) method was carried out as described (Abdul Rahman et al., 2014). Briefly, 1 × 10^6^ cells were seeded into a 10 cm culture dish and harvested 24 h later for glycan preparation. Following SDS lysis and buffer exchange in a centrifugal filter, glycans were released using PNGase F treatment, and permethylated prior to mass spectrometric analysis.

### Mass spectrometry

Permethylated glycans were dissolved in 20 μL methanol. Two microliters of this sample was mixed with 1 μL of 0.5 M sodium nitrate (in 70% methanol) and 2 μL of 20 mg/mL 2,5-dihydroxybenzoic acid (in 70% methanol). Two microliters of this mix was spotted onto a ground steel MALDI target plate (Bruker) and allowed to air dry. Immediately afterwards, 0.2 μL of ethanol was added to the spot and left to air dry for re-crystallization. Samples were analyzed using an ultraflex III MALDI-TOF mass spectrometer (Bruker). The spectra were acquired over the *m/z* range 800–6000 using positive-ion mode, with 4000 laser shots in steps of 800, which were summed to give one spectrum per spot. The Smartbeam™ laser power was set to 50–65%.

### Data analysis

Spectra were analyzed using flexAnalysis 3.3 (Bruker), first processed using the centroid peak detection algorithm, using a signal to noise limit of 3, and smoothed for 1 cycle at 0.1 *m/z*, using the Savitzky-Golay algorithm. Glycan peaks were identified from their mono-isotopic peak *m/z* values on comparison with those for established *N*-glycan structures. For a glycan to be included in the analysis at least two of its isotopic peaks had to have intensities >3x the noise, and the associated peaks had to fall into an isotopic pattern as predicted based on the glycan's atomic composition (Bruker Compass's Isotope Pattern function). The peak intensities of all isotope signals >3x noise for a given glycan were summed to provide that glycan's total peak intensity. To compare different spectra, the total peak intensity values were normalized to the total peak intensities of all glycans identified in that spectrum. Normalized peak intensities could then be averaged across different spectra of the same sample type.

### Flow cytometry analysis

Cells were washed twice with PBS, and incubated with washing buffer (0.2% bovine serum albumin, 5 mM EDTA in PBS) at 37°C until cells detached. Cells were centrifuged for 5 min at 450 g, resuspended in PBS and 150,000 cells pelleted and resuspended in 120 μL ice-cold PBS. Following 15 min incubation on ice, 120 μL of 10 μg/mL FITC-ConA (Vector Labs) was added to each sample. After two 15 min incubations interspersed with flicking of the tubes, 1 mL of washing buffer was added and the samples were centrifuged for 5 min at 450 g. The cell pellet was resuspended in 100 μL of washing buffer containing 1 μg/mL DAPI and incubated on ice in the dark for 5 min, followed by the addition of 1 mL washing buffer, and centrifugation for 5 min at 450 g. The pellet was resuspended in 400 μL PBS for flow cytometry. Data were gated for DAPI-negative FITC-positive cells. Median fluorescence intensity was determined for all gated cells in the sample.

Analysis of cell surface markers was performed as previously described (James et al., 2015).

### Statistics

Data were analyzed using Sigma Plot 12.3. Before statistical tests were applied, a normality test (Shapiro–Wilk) and a test of equal variance was performed. For comparing two groups if data passed, a Student's *T*-test was carried out. Otherwise, one-way ANOVA tests were carried out, followed by Holm Sidak *post-hoc* tests if required. If the data failed the normality or variance tests, equivalent non-parametric tests were applied instead. Either a Mann–Whitney test when two groups were being compared, or a Kruskal–Wallis one-way ANOVA on ranks, followed by Dunn's *post-hoc* tests was carried out if more than one group was being compared. Throughout ^*^:*P* < 0.05, ^**^:*P* < 0.01, and ^***^:*P* < 0.001.

## Results

### Osteogenic differentiation of Y101 cells alters their *N*-glycan profile

**Figure 1:**
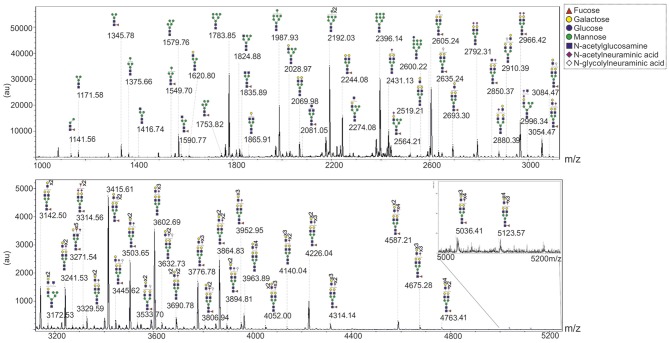
*****N***-glycan profile of Y101 hTERT-MSC line**. Representative MALDI-TOF spectrum of permethylated *N*-glycans isolated from the Y101 hTERT-MSC line. Peaks were annotated based on the calculated glycan mass and knowledge of the glycan biosynthesis pathway. *m/z* value of the mono-isotopic peak is labeled, with the predicted structure pictured above. Spectrum shown is from *m/z* 1000 to 5250 split into two sections. Note that the sections are plotted on different y axis scales to make the higher *m/z* range section easier to see.

To study the sub-type-specific properties of cells from a primary human MSC mixture, previously generated telomerase immortalized lines derived from primary human MSCs, termed hTERT-MSCs (James et al., 2015), were used. The *N*-glycans of one of these lines, Y101, were profiled using the FANGS method (Abdul Rahman et al., 2014). Figure 1 shows a typical *N*-glycan profile of Y101 cells in which as many as 65 different glycan species were detected. The glycan profile is qualitatively comparable with those reported for primary MSCs (Heiskanen et al., 2009; Hamouda et al., 2013), although different glycan classes cannot be quantitatively compared with those reported in the older studies, due to their different analysis strategies. The most abundant glycans we observe in these cells are the Man_6_-Man_9_ oligomannose species (*m/z* 1783.85, 1987.93, 2192.03, and 2396.14). Among the complex glycans, the singly sialylated, fucosylated biantennary species (*m/z* 2605.24), followed closely by the same glycan without sialic acid (*m/z* 2244.08) and then the bis-sialylated version (*m/z* 2966.42) are most abundant. It is worth noting that the majority of the large complex glycans are fucosylated (e.g., *m/z* 4226.04, 4314.14, 4587.21, 4676.28, 4764.41, 5037.41, and 5124.57), which most likely indicates the presence of core fucose.

**Figure 2:**
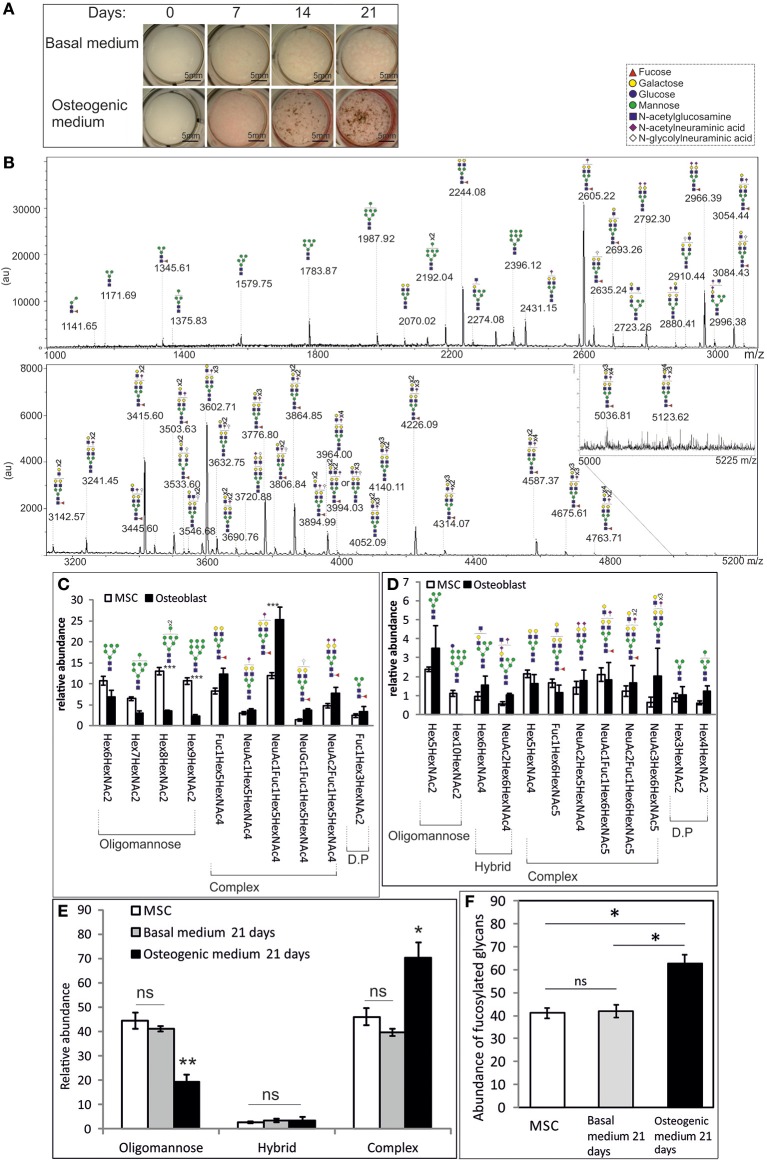
**Osteogenic differentiation significantly alters the ***N***-glycan profile of MSCs. (A)** Brightfield images of Y101 hTERT-MSCs cultured in basal **(top row)** or osteogenic **(bottom row)** media, stained for alkaline phosphatase (pink) and phosphate (von Kossa, brown), sampled at weekly intervals. **(B)** A representative MALDI-TOF spectrum of permethylated *N*-glycans isolated from osteoblasts cultured for 21 days as in **(A)**. Presented as in Figure 1. **(C–F)** Comparisons of averaged (Y101 *n* = 5, osteoblast *n* = 3) normalized total peak intensities of: **(C)** Individual glycan structures with abundances above 3% of the total. **(D)** Individual glycan structures with abundances of 1–3% of the total. D.P, degradation product, most likely produced in the lysosome. **(E)** Sums of different glycan types. **(F)** Sums of all fucosylated glycan abundances. Error bars show standard error of the mean. In **(E, F)** the “Basal medium 21 days” sample was harvested following culture in basal medium for 21 days (*n* = 2). For a representative spectrum of this sample see Supplementary Figure 1. ^*^P < 0.05, ^**^P < 0.01, ^***^P < 0.001.

Changes in the *N*-glycan profile of primary MSCs upon differentiation have been reported (Heiskanen et al., 2009), again with the caveat that that analysis did not allow detailed quantification. The telomerase-immortalized Y101 cell line is capable of robust osteogenic differentiation *in vitro* (James et al., 2015). This is demonstrated when the cells are stained for secreted alkaline phosphatase (ALP, pink staining) and hydroxyapatite mineral deposits (brown) with an ALP/von Kossa stain at 7 day intervals over a 3 week period (Figure 2A). It is clear from appearance of the pink ALP stain and the stained phosphate deposits that 21 days in osteogenic medium gives strong differentiation (Figure 2A bottom row “21 days” image). These differentiated cells were harvested and their total *N*-glycan repertoire analyzed using FANGS followed by MALDI-TOF-MS (Figure 2B). The resulting spectra show a very different picture than that from the undifferentiated MSCs. Importantly, the observed changes in glycosylation during osteogenesis were not due to the 21 days spent in culture. When Y101 MSCs were cultured in basal rather than osteogenic medium for 21 days (a treatment that does not promote osteogenesis) the *N*-glycan spectra obtained were similar to those from MSCs at the start of the incubation period (Figures 2E,F and Supplementary Figure 1). In the differentiated osteoblasts, by far the most abundant glycan peak is now the singly sialylated, fucosylated biantennary complex glycan species (*m/z* 2605.22), followed, at about half the intensity, by the same complex glycan without sialic acid (*m/z* 2244.08) and the fully sialylated species (*m/z* 2966.39). The most abundant oligomannose glycan in osteoblasts, the Man_6_ species (*m/z* 1783.87), is only ranked fourth in relative intensity in the *N*-glycome of osteoblasts (Figure 2B, Table 1).

**Table 1:** **Averaged relative abundances of individual ***N***-glycan structures isolated from Y101 hTERT-MSCs and their osteoblast progeny as well as MSCs kept for 21 days in basal medium**.

**Glycan composition**	**Type^a^**	**Average relative abundance**^**b**^		
		**MSC**	**Osteoblast**	**21 day basal**
Hex_5_HexNAc_2_	O	2.394 ± 0.120	3.505 ± 1.182	7.095 ± 1.526
Fuc_1_Hex_5_HexNAc_2_	O	0.621 ± 0.215	0.187 ± 0.187	0.885 ± 0.405
Hex_6_HexNAc_2_	O	10.740 ± 0.998	6.846 ± 1.639	13.342 ± 2.521
Hex_7_HexNAc_2_	O	6.470 ± 0.480	3.010 ± 0.499	6.258 ± 1.252
Hex_8_HexNAc_2_	O	13.042 ± 0.894	3.476 ± 0.160	9.089 ± 0.790
Hex_9_HexNAc_2_	O	10.693 ± 0.825	2.383 ± 0.345	5.174 ± 0.521
Hex_10_HexNAc_2_	O	1.119 ± 0.154	0	0.186 ± 0.062
Hex_5_HexNAc_3_	H	0.584 ± 0.114	0.509 ± 0.328	0.615 ± 0.166
Hex_6_HexNAc_3_	H	0.212 ± 0.130	0.638 ± 0.370	0.916 ± 0.192
Hex_6_HexNAc_4_	H	0.965 ± 0.217	1.557 ± 0.483	1.246 ± 0.351
NeuAc_1_Hex_6_HexNAc_3_	H	0.432 ± 0.184	0.594 ± 0.302	0.709 ± 0.090
NeuAc_1_Fuc_1_Hex_6_HexNAc_3_	H	0.032 ± 0.032	0	0
Hex_7_HexNAc_5_	H	0.438 ± 0.127	0.207 ± 0.089	0.125 ± 0.040
NeuAc_2_Hex_6_HexNAc_4_	H	0.581 ± 0.108	1.032 ± 0.084	0.304 ± 0.269
Hex_8_HexNAc_6_	H	0.066 ± 0.031	0	0
Hex_3_HexNAc_3_	C	0.004 ± 0.004	0.178 ± 0.178	0.091 ± 0.112
Fuc_1_Hex_3_HexNAc_3_	C	0.431 ± 0.084	0.185 ± 0.185	0.292 ± 0.195
Hex_4_HexNAc_3_	C	0.335 ± 0.085	0.197 ± 0.197	0.323 ± 0.112
Fuc_1_Hex_3_HexNAc_4_	C	0.686 ± 0.106	0.126 ± 0.126	0.287 ± 0.101
Hex_4_HexNAc_4_	C	0.285 ± 0.056	0.089 ± 0.089	0.224 ± 0.134
Fuc_1_Hex_4_HexNAc_4_	C	0.552 ± 0.119	0	0
Hex_5_HexNAc_4_	C	2.151 ± 0.214	1.622 ± 0.485	3.370 ± 0.447
Fuc_1_Hex_3_HexNAc_5_	C	0.297 ± 0.093	0	0.000 ± 0.138
NeuAc_1_Fuc_1_Hex_4_HexNAc_3_	C	0.379 ± 0.104	0.139 ± 0.139	0.597 ± 0.269
Fuc_1_Hex_5_HexNAc_4_	C	8.259 ± 0.655	12.271 ± 1.386	11.951 ± 2.082
NeuAc_1_Fuc_1_Hex_5_HexNAc_3_	C	0	0.138 ± 0.138	0.337 ± 0.132
NeuAc_1_Hex_5_HexNAc_4_	C	3.040 ± 0.347	3.757 ± 0.367	3.330 ± 0.169
Hex_6_HexNAc_5_	C	0.383 ± 0.123	0.088 ± 0.088	0.204 ± 0.045
NeuAc_1_Fuc_1_Hex_5_HexNAc_4_	C	11.968 ± 0.722	25.387 ± 2.830	12.152 ± 4.007
NeuGc_1_Fuc_1_Hex_5_HexNAc_4_	C	1.348 ± 0.273	3.712 ± 0.423	1.346 ± 0.789
Fuc_1_Hex_6_HexNAc_5_	C	1.661 ± 0.213	1.171 ± 0.382	0.590 ± 0.184
NeuAc_2_Hex_5_HexNAc_4_	C	1.421 ± 0.340	1.808 ± 0.547	0.619 ± 0.078
NeuAc_1_Fuc_1_Hex_5_HexNAc_5_	C	0.143 ± 0.059	0	0
NeuAc_1_Hex_6_HexNAc_5_	C	0.461 ± 0.101	0.314 ± 0.150	0.125 ± 0.033
NeuGc_1_Hex_6_HexNAc_5_	C	0.252 ± 0.052	0.140 ± 0.112	0.040 ± 0.032
NeuAc_2_Fuc_1_Hex_5_HexNAc_4_	C	4.784 ± 0.583	7.695 ± 1.401	2.957 ± 1.398
NeuAc_1_Fuc_1_Hex_6_HexNAc_5_	C	2.102 ± 0.360	1.827 ± 0.904	0.599 ± 0.323
NeuGc_1_Fuc_1_Hex_6_HexNAc_5_	C	0.351 ± 0.086	0.241 ± 0.103	0.071 ± 0.044
Fuc_1_Hex_7_HexNAc_6_	C	0.398 ± 0.084	0.134 ± 0.082	0.043 ± 0.028
NeuAc_2_Hex_6_HexNAc_5_	C	0.248 ± 0.096	0.274 ± 0.050	0.249 ± 0.238
NeuAc_1_Fuc_6_Hex_4_HexNAc_4_	C	0.025 ± 0.016	0	0
NeuAc_2_Fuc_3_Hex_5_HexNAc_4_	C	0.011 ± 0.011	0	0
NeuAc_1_Hex_7_HexNAc_6_	C	0.091 ± 0.030	0	0
NeuAc_2_Fuc_1_Hex_6_HexNAc_5_	C	1.228 ± 0.269	1.670 ± 0.932	0.345 ± 0.328
NeuAc_1_NeuGc_1_Fuc_1_Hex_6_HexNAc_5_	C	0.126 ± 0.036	0.210 ± 0.097	0.037 ± 0.057
NeuAc_1_Fuc_1_Hex_7_HexNAc_6_	C	0.588 ± 0.147	0.321 ± 0.242	0.058 ± 0.049
NeuAc_1_Hex_8_HexNAc_6_	C	0.075 ± 0.029	0.042 ± 0.042	0.000 ± 0.013
NeuGc_2_Hex_6_HexNAc_6_	C	0.014 ± 0.014	0.058 ± 0.058	0
NeuAc_3_Hex_6_HexNAc_5_	C	0.657 ± 0.268	2.034 ± 1.445	0.027 ± 0.033
NeuAc_2_NeuGc_1_Hex_6_HexNAc_5_	C	0.045 ± 0.027	0.288 ± 0.194	0
NeuAc_2_Hex_7_HexNAc_6_	C	0.077 ± 0.041	0.092 ± 0.092	0
NeuAc_1_NeuGc_1_Hex_7_HexNAc_6_	C	0	0.054 ± 0.054	0
NeuAc_3_Fuc_1_Hex_6_HexNAc_5_	C	0.373 ± 0.114	0.993 ± 0.611	0.089 ± 0.135
NeuAc_2_NeuGc_1_Fuc_1_Hex_6_HexNAc_5_	C	0.036 ± 0.015	0.167 ± 0.100	0.014 ± 0.028
NeuAc_2_Fuc_1_Hex_7_HexNAc_6_	C	0.520 ± 0.176	0.735 ± 0.587	0.068 ± 0.104
NeuAc_1_NeuGc_1_Fuc_1_Hex_7_HexNAc_6_	C	0.038 ± 0.025	0.088 ± 0.076	0.009 ± 0.018
NeuAc_1_Fuc_1_Hex_8_HexNAc_7_	C	0.047 ± 0.030	0	0
NeuAc_4_Hex_6_HexNAc_5_	C	0.083 ± 0.043	0.377 ± 0.255	0
NeuAc_1_Fuc_1_Hex_7_HexNAc_8_or^c^ NeuAc_3_NeuGc_1_Hex_6_HexNAc_5_	C	0	0.065 ± 0.040	0
NeuAc_3_Hex_7_HexNAc_6_	C	0.023 ± 0.014	0.037 ± 0.037	0
NeuAc_2_Hex_8_HexNAc_7_	C	0.004 ± 0.004	0.024 ± 0.024	0
NeuAc_1_NeuGc_1_Hex_8_HexNAc_7_ or^c^ NeuGc_2_Fuc_1_Hex_7_HexNAc_7_	C	0	0.016 ± 0.016	0
NeuAc_3_Fuc_1_Hex_7_HexNAc_6_	C	0.174 ± 0.077	0.427 ± 0.332	0.024 ± 0.057
Fuc_2_Hex_8_HexNAc_9_ or^c^ NeuAc_3_Hex_8_HexNAc_6_	C	0	0.057 ± 0.047	0.000 ± 0.009
NeuAc_2_Fuc_1_Hex_8_HexNAc_7_	C	0.033 ± 0.020	0.070 ± 0.060	0.000 ± 0.006
NeuAc_4_Fuc_1_Hex_7_HexNAc_6_	C	0.046 ± 0.024	0.233 ± 0.177	0.005 ± 0.017
NeuAc_3_Fuc_1_Hex_8_HexNAc_7_	C	0.016 ± 0.010	0.061 ± 0.051	0
NeuAc_2_Fuc_1_Hex_9_HexNAc_8_	C	0.008 ± 0.005	0.013 ± 0.013	0
NeuAc_4_Fuc_1_Hex_8_HexNAc_7_	C	0.002 ± 0.002	0.019 ± 0.014	0
NeuAc_3_Fuc_1_Hex_9_HexNAc_8_	C	0.001 ± 0.001	0.011 ± 0.011	0
Fuc_1_Hex_2_HexNAc_2_	DP^d^	0.745 ± 0.190	0.746 ± 0.221	1.955 ± 0.311
Hex_3_HexNAc_2_	DP	0.891 ± 0.220	1.053 ± 0.422	2.830 ± 0.603
Fuc_1_Hex_3_HexNAc_2_	DP	2.402 ± 0.504	3.291 ± 1.230	6.714 ± 1.163
Hex_4_HexNAc_2_	DP	0.618 ± 0.106	1.216 ± 0.282	1.641 ± 0.323
Fuc_1_Hex_4_HexNAc_2_	DP	0.396 ± 0.095	0	0.571 ± 0.227

a *Glycans grouped as oligomannose (O), hybrid (H), complex (C), and degradation product (DP)*.

b *Percentage of total glycan signal ± SEM for n = 5 (MSC), n = 3 (osteoblast) and n = 2 (21 day basal) are shown*.

c *Given the mass of these glycans other glycan compositions are possible, but the most plausible two possibilities are shown*.

d *DP are small glycans that are not produced by the mammalian N-glycosylation machinery and therefore most likely originate from lysosomal degradation*.

Averaging the glycan peak intensities from several biological replicates showed that the most abundant complex glycans were indeed reproducibly much more abundant in osteoblasts than undifferentiated MSCs (Figure 2C, Table 1). In contrast, the large oligomannose glycans all had very low abundances in the *N*-glycomes of osteoblasts, although these are among the most abundant glycans in those of the MSCs (Figure 2C, Table 1). Similarly, the singly glucosylated oligomannose glycan species was undetectable from osteoblasts, although it was notably present from MSCs (*m/z* 2600.22 Figures 1, 2D, Table 1). There was also a trend toward increased abundance of hybrid glycans in osteoblasts (Figure 2D, Table 1). Differences are even more evident when the total oligomannose and complex glycan levels are compared. The reduction in relative levels of oligomannose glycans and the increase in complex glycans are both highly significant (Figure 2E). There is also a significant increase in the total amount of fucosylated species detected from the osteoblasts over that from their MSC progenitors (Figure 2F), although glycan species with multiple fucoses were more prevalent in the MSCs (Table 1).

### MSC lines share a similar *N*-glycan profile independent of their altered differentiation potentials

**Figure 3:**
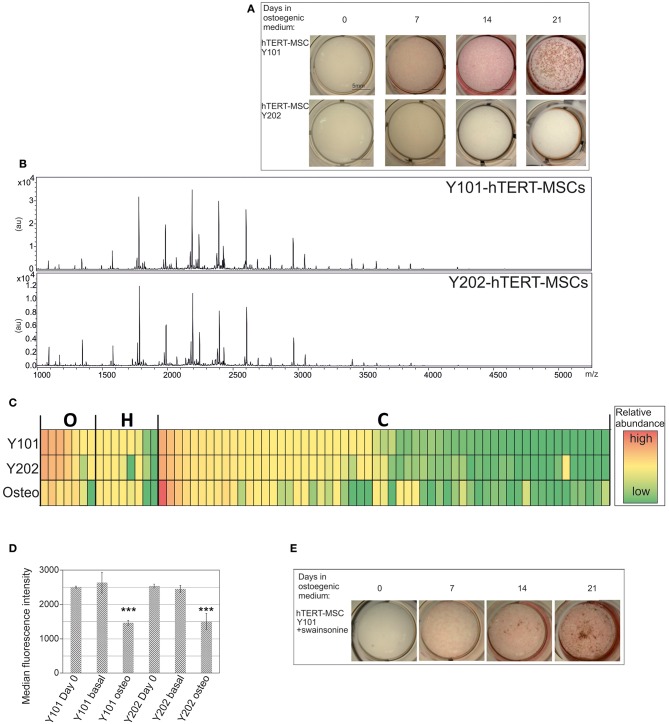
*****N***-glycan profile of MSCs does not correlate with differentiation capacity. (A)** Osteogenic capacities of Y101 and Y202 hTERT-MSC lines. For details see Figure 2A. **(B)** *N*-glycan profiles of the Y101 and Y202 hTERT-MSC lines following 24 h culture in basal medium. Representative MALDI-TOF spectra as in Figure 1, but glycan assignments are omitted for clarity. **(C)** Heat map display comparing the *N*-glycan profiles of Y101, Y202 hTERT-MSCs, and osteoblasts derived from the Y101 hTERT-MSCs. Averaged (Y101, Y202 *n* = 5, osteoblasts *n* = 3) normalized peak intensities were compared. Each row in the heat map display represents a single glycan structure. Glycans are grouped by type: oligomannose (O), hybrid (H), or complex (C), and sorted by abundance in the Y101 profile within each type. The cells' colors denote glycan abundance as indicated in the legend. **(D)** Y101 or Y202 cells were grown for 0 or 8 days in basal or osteogenic (osteo) medium, then single cell suspensions stained with ConA-FITC and DAPI and analyzed by flow cytometry. The median intensity of the FITC fluorescence of live cells is shown with SEM from three independent replicates. **(E)** Y101 cells were cultured in basal medium containing 10 μg/mL swainsonine for 48 h before addition of osteogenic medium containing swainonsine at the same concentration. Mineral deposition and ALP activity were tested by ALP/von Kossa staining 0, 7, 14, and 21 days after addition of osteogenic medium. ^***^P < 0.001.

Given that differentiation considerably alters the *N*-glycan profile, we wondered whether MSCs with diverse differentiation abilities present variations in *N*-glycan profiles. We compared two hTERT-MSC lines: Y101 and Y202. Y101 cells differentiate well into osteoblasts. Y202 cells in contrast, which do show the cell surface markers characteristic of MSCs (Supplementary Figure 3; Dominici et al., 2006), are virtually devoid of differentiation potential (James et al., 2015) as exemplified by the lack of ALP and von Kossa staining, used to detect osteogenesis (Figure 3A bottom row).

To assess if the differences in differentiation capacity between the different clonal MSC lines are associated with a variation in their *N*-glycan repertoire, we used FANGS followed by MALDI-TOF-MS to profile their glycans. Both MSC lines were cultured for 24 h in basal medium post passaging, prior to *N*-glycan sample harvest and analysis. The overall *N*-glycan profiles of these lines look very similar to each other (Figure 3B). Profiling of several biological replicates for the same cell lines followed by averaging of the relative glycan abundances revealed no significant differences at all (Supplementary Figures 2A,B and Supplementary Table 1). This lack of difference is also apparent when comparing different glycan classes (Supplementary Figures 2C,D). A heat map generated using the averaged relative abundances of all glycans from the two different MSC lines, Y101 and Y202, and the osteoblasts derived from Y101 cells clearly shows that the main change in *N*-glycan profile is between the MSCs and the differentiated progeny, rather than within the different lines of MSCs (Figure 3C).

It is clear that differentiation alters the glycan profile, yet the glycan profile of the stem cells does not provide clues to their differentiation potential. We therefore wondered if the glycosylation changes are restricted to cells undergoing differentiation, or whether non-differentiating cells, when incubated under differentiation-promoting conditions, alter their glycan profiles. The reduction of oligomannose glycan content was investigated for MSCs incubated in osteogenic medium for 8 days. As expected, Y101 cells showed a clear reduction in oligomannose content, as evidenced by reduced ConA-FITC staining measured using flow cytometry when compared with cells cultured in basal medium (Figure 3D, second and third bars). The lectin ConA binds oligomannose glycans, and is therefore a sensitive tool for uncovering changes in the proportion of this glycan class. Surprisingly, Y202 cells, which do not form mineral deposits, showed the same highly significant reduction in oligomannose content when incubated in osteogenic medium (Figure 3D, fifth and sixth bars). Thus although Y202 cells do not differentiate into mineral-depositing osteoblasts, they do undergo a significant change, which results in a glycan profile alteration similar to that of osteoblastic Y101 cells.

As a second test we wondered if glycosylation changes are necessary for differentiation into osteoblasts to take place. We used the glycan-processing inhibitor swainsonine, which inhibits mannosidase II. As expected, this caused a major shift in the glycan profile, in which complex glycans almost completely disappeared, and were replaced by hybrid ones (Supplementary Figure 4). This allowed us to test whether the shift to complex glycans is required for osteogenesis. While there was a slight reduction in the amount of mineral deposition when the cells where grown in osteogenic medium in the presence of swainsonine, the cells were clearly still capable of forming osteoblasts and depositing calcium phosphate mineral (Figure 3E).

## Discussion

A previous glycomic study concluded that oligomannose glycans are more abundant in parental stem cells than in their differentiated osteoblasts (Heiskanen et al., 2009). As the glycans in that study were not permethylated a true quantitative assessment of the relative abundance of the various glycan species was not possible (Wada et al., 2007). Our study now provides a detailed quantitative comparison of the MSC and osteoblast glycan profiles of clonal lines made possible by the FANGS method. We find that oligomannose and complex glycans are close to equally abundant in MSCs, while this changes to an almost four-fold higher abundance of the complex glycans in osteoblasts. Shifts in the glycan profile upon differentiation have been reported using quantitative methods such as the MALDI-MS of permethylated glycans and LC-MS of fluorescently labeled glycans (Wada et al., 2007; Hasehira et al., 2012). Comparing neuronal stem cells and their differentiated progeny, (Yagi et al., 2012) or embryonic stem cells and embryoid bodies (Nairn et al., 2012), showed reductions in oligomannose glycans in the differentiated cells. Along similar lines, the reverse process, generation of induced pluripotent stem (iPS) cells, increased the oligomannose proportion of the glycan profile (Hasehira et al., 2012). None of these comparisons reported a shift in the proportion of oligomannose vs. complex glycans as large as that observed in our study. A likely explanation is that most studies have not compared terminally differentiated specialized cells, but intermediate or mixed stages of differentiation. This possibility is corroborated by the glycomic profiles published for embryonic stem cells and terminally differentiated fibroblasts (An et al., 2012). This latter study used clonal lines, just as our study does, but did not use permethylated glycans, so their quantification is imperfect. Moreover, the fibroblasts investigated were not derived from the stem cells analyzed, so the comparison is largely between a generic stem cell and a generic differentiated cell population, rather than the consequence of the differentiation process *per se* as in our study. Nevertheless, the shift in oligomannose glycan abundance from 70 to 80% in the stem cells to 30% in the fibroblasts (An et al., 2012) is close to the range that we now report. Our results would therefore be in line with the hypothesis that stemness may require oligomannose glycans (Heiskanen et al., 2009; Hamouda et al., 2013).

Another change within the glycan profile is increased overall fucosylation intensity in osteoblasts. Single fucosylation could be present either on the core or the antennae, but glycans with multiple fucoses have to contain fucose on the antennae. From MSCs we observed several glycans with multiple fucoses as opposed to osteoblasts where single fucosylation predominates. Thus while the overall intensity of fucosylation is elevated in osteoblasts, the level of fucosylation on antennae is likely higher in the stem cells, leading us to speculate that fucosylation on the antennae could be a property of stem cells rather than osteoblasts (Figures 1, 2B and Table 1). Indeed, iPS cells have been shown to have a lower degree of core fucosylation but more on the antennae than their differentiated parents (Hasehira et al., 2012). In addition, Fut9-dependent fucose addition to the antennae has been implicated in embryonic stem cell self-renewal (Li et al., 2009).

Comparison of the two MSC lines shows a remarkable similarity in their *N*-glycan profiles, despite a marked difference in their differentiation capabilities. The profiles of the stem cell lines we report are also similar to the previously reported profiles of primary MSCs (Heiskanen et al., 2009; Hamouda et al., 2013). We would therefore argue that *N*-glycan profiles are a good indicator of overall MSC identity. Importantly, changes to the glycan profile following prolonged culturing of the stem cells are also small, enforcing the usefulness of glycans as an MSC marker. The main difference between the glycan profiles of MSCs before and after prolonged culturing is a significantly higher abundance of degradation products (Table 1). The appearance of such *N*-glycans that cannot be made during biosynthetic processing in mammals is most likely a consequence of intermediates accumulating in the lysosome during glycan degradation (Uematsu et al., 2005). These are picked up in our analysis due to the FANGS process capturing all cellular protein-linked *N*-glycans. The amount of these glycan species in the MSCs and osteoblasts is in line with the levels of paucimannose glycans found in other glycan profiling studies of cultured cells (Abdul Rahman et al., 2014). The increased proportion of this glycan class in the cells that were cultured for 21 days may be explained by increased autophagy and/or cell death, possible consequences of over-confluency due to the lack of differentiation. A further conclusion we can make from these observations is that the differences between MSC and osteoblast glycan profiles are not due to the extended culture time, but rather are genuine differentiation-associated changes.

A major question in glycobiology is the function of glycans during cellular processes. While it is well documented that there are changes in glycan profiles between cell types, the functional consequence of these changes is unclear. Our results suggest that the shift to complex *N*-glycans in MSCs is neither required nor sufficient alone to drive osteogenesis. Inhibition of complex *N*-glycan formation by swainsonine does not inhibit osteogenesis. In contrast, the shift to a lower proportion of oligomannose glycans in Y202 cells is not accompanied by osteoblast mediated mineralization. Rather, it could more likely be the stem-like properties of MSCs that may benefit from certain glycan features.

Our detailed analysis of two MSC cell lines allows us to speculate about the requirements for the various glycan subtypes of different stem cell functions. Given that the Y202 line, which is unable to differentiate effectively, has the same *N*-glycan profile as the differentiating Y101 MSC line, it is unlikely that glycans directly determine the differentiation potential of these cells. However, continued proliferation and self-renewal are also very important properties of stem cells. Indeed terminal fucosylation has been suggested to be an important factor in stem cell maintenance (Li et al., 2009). It is therefore a possibility that some of the features of the profiles found in both the differentiating and undifferentiating MSC lines, which are absent from osteoblasts, such as increased oligomannose content or the fucosylation of antennae, may contribute to the maintenance of a proliferative phenotype.

## Author contributions

KW, PG, and DU designed the study; KW performed the experiments; KW, JTO, and DU analyzed the data; KW, JTO, PG, and DU wrote the paper.

### Conflict of interest statement

The authors declare that the research was conducted in the absence of any commercial or financial relationships that could be construed as a potential conflict of interest.

## Supplementary material

Supplementary Table 1 **Comparison of averaged glycan abundances for the hTERT-MSC lines Y101 and Y202**.

Supplementary Figure 1 **Representative MALDI-TOF spectrum of permethylated *N*-glycans harvested from MSCs grown for 21 days in basal medium**. For details see Figure 1.

Supplementary Figure 2 **Comparison of Y101 and Y202 *N*-glycans, for details see Figures 2C–F**. Comparisons of averaged (*n* = 5) Y101 and Y202 normalized *N*-glycan total peak intensities of: **(A)** Individual glycan structures with abundances above 3% of the total. **(B)** Individual glycan structures with abundances of 1–3% of the total. D.P, degradation product, most likely produced in the lysosome. **(C)** Sums of different glycan types. **(F)** Sums of all fucosylated glycan abundances. Error bars show standard error of the mean.

Supplementary Figure 3 **Presence and absence of the indicated cell surface CD markers was determined for Y202 cells using flow cytometry**.

Supplementary Figure 4 **Comparison of representative MALDI-TOF spectra of *N*-glycans harvested from Y101 MSCs cultured for 48 h in the presence or absence of swainsonine**. The assigned glycan structures are only shown for the most prominent peaks.

## Abbreviations

ALP: Alkaline phosphatase
FANGS: Filter-aided *N*-glycan separation
MALDI: Matrix-assisted laser desorption/ionization
MS: Mass spectrometry
MSC: Mesenchymal stromal cell
SEM: Standard error of the mean
TOF: Time-of-flight.
