# Supplementary material for: Glycan Profiling Shows Unvaried N-Glycomes in MSC Clones with Distinct Differentiation Potentials
Source: Front Cell Dev Biol. 2016 May 31;4:52. doi: 10.3389/fcell.2016.00052 (PMC4885867; doi:10.3389/fcell.2016.00052)
Supplement: Supplementary Table 1 — Comparison of averaged glycan abundances for the hTERT-MSC lines Y101 and Y202. [file Table1.DOCX]

**Supplementary Table I.**

**Comparison of averaged glycan abundances for the hTERT-MSC lines Y101 and Y202**

| **Glycan composition** | **Type^a^** | **Average relative abundance^b^** | |
| --- | --- | --- | --- |
|  |  | **Y101** | **Y202** |
| Hex_5_HexNAc_2_ | O | 2.394 ± 0.120 | 2.225 ± 0.472 |
| Fuc_1_Hex_5_HexNAc_2_ | O | 0.621 ± 0.215 | 0.132 ± 0.132 |
| Hex_6_HexNAc_2_ | O | 10.740 ± 0.998 | 9.803 ± 1.599 |
| Hex_7_HexNAc_2_ | O | 6.470 ± 0.480 | 6.018 ± 0.800 |
| Hex_8_HexNAc_2_ | O | 13.042 ± 0.894 | 11.945 ± 1.464 |
| Hex_9_HexNAc_2_ | O | 10.693 ± 0.825 | 9.490 ± 1.059 |
| Hex_10_HexNAc_2_ | O | 1.119 ± 0.154 | 0.937 ± 0.136 |
|  |  |  |  |
| Hex_5_HexNAc_3_ | H | 0.584 ± 0.114 | 0.520 ± 0.121 |
| Hex_6_HexNAc_3_ | H | 0.212 ± 0.130 | 0.373 ± 0.113 |
| Hex_6_HexNAc_4_ | H | 0.965 ± 0.217 | 1.155 ± 0.321 |
| NeuAc_1_Hex_6_HexNAc_3_ | H | 0.432 ± 0.184 | 0.206 ± 0.130 |
| NeuAc_1_Fuc_1_Hex_6_HexNAc_3_ | H | 0.032 ± 0.032 | 0 |
| Hex_7_HexNAc_5_ | H | 0.438 ± 0.127 | 0 |
| NeuAc_2_Hex_6_HexNAc_4_ | H | 0.581 ± 0.108 | 0.829 ± 0.262 |
| Hex_8_HexNAc_6_ | H | 0.066 ± 0.031 | 0.117 ± 0.048 |
|  |  |  |  |
| Hex_3_HexNAc_3_ | C | 0.004 ± 0.004 | 0.016 ± 0.016 |
| Fuc_1_Hex_3_HexNAc_3_ | C | 0.431 ± 0.084 | 0.622 ± 0.132 |
| Hex_4_HexNAc_3_ | C | 0.335 ± 0.085 | 0.373 ± 0.095 |
| Fuc_1_Hex_3_HexNAc_4_ | C | 0.686 ± 0.106 | 0.683 ± 0.105 |
| Hex_4_HexNAc_4_ | C | 0.285 ± 0.056 | 0.192 ± 0.081 |
| Fuc_1_Hex_4_HexNAc_4_ | C | 0.552 ± 0.119 | 0.132 ± 0.062 |
| Hex_5_HexNAc_4_ | C | 2.151 ± 0.214 | 2.014 ± 0.237 |
| Fuc_1_Hex_3_HexNAc_5_ | C | 0.297 ± 0.093 | 0.247 ± 0.135 |
| Fuc_1_Hex_5_HexNAc_4_ | C | 8.259 ± 0.655 | 8.243 ± 0.911 |
| NeuAc_1_Fuc_1_Hex_4_HexNAc_3_ | C | 0.379 ± 0.104 | 0.776 ± 0.133 |
| NeuAc_1_Hex_5_HexNAc_4_ | C | 3.040 ± 0.347 | 3.067 ± 0.441 |
| NeuAc_1_Hex_4_HexNAc_5_ | C | 0 | 0.444 ± 0.090 |
| Hex_6_HexNAc_5_ | C | 0.383 ± 0.123 | 0.283 ± 0.127 |
| NeuAc_1_Fuc_1_Hex_5_HexNAc_4_ | C | 11.968 ± 0.722 | 12.517 ± 1.230 |
| NeuGc_1_Fuc_1_Hex_5_HexNAc_4_ | C | 1.348 ± 0.273 | 1.783 ± 0.537 |
| Fuc_1_Hex_6_HexNAc_5_ | C | 1.661 ± 0.213 | 2.147 ± 0.311 |
| NeuAc_2_Hex_5_HexNAc_4_ | C | 1.421 ± 0.340 | 1.364 ± 0.256 |
| NeuAc_1_Fuc_1_Hex_5_HexNAc_5_ | C | 0.143 ± 0.059 | 0.066 ± 0.032 |
| NeuAc_1_Hex_6_HexNAc_5_ | C | 0.461 ± 0.101 | 0.404 ± 0.076 |
| NeuGc_1_Hex_6_HexNAc_5_ | C | 0.252 ± 0.052 | 0.207 ± 0.051 |
| NeuAc_2_Fuc_1_Hex_5_HexNAc_4_ | C | 4.784 ± 0.583 | 5.372 ± 0.795 |
| NeuAc_1_Fuc_1_Hex_6_HexNAc_5_ | C | 2.102 ± 0.360 | 2.282 ± 0.363 |
| NeuGc_1_Fuc_1_Hex_6_HexNAc_5_ | C | 0.351 ± 0.086 | 0.388 ± 0.084 |
| Fuc_1_Hex_7_HexNAc_6_ | C | 0.398 ± 0.084 | 0.626 ± 0.116 |
| NeuAc_2_Hex_6_HexNAc_5_ | C | 0.248 ± 0.096 | 0.274 ± 0.050 |
| NeuAc_1_Fuc_6_Hex_4_HexNAc_4_ | C | 0.025 ± 0.016 | 0.036 ± 0.017 |
| NeuAc_2_Fuc_3_Hex_5_HexNAc_4_ | C | 0.011 ± 0.011 | 0.047 ± 0.025 |
| NeuAc_1_Hex_7_HexNAc_6_ | C | 0.091 ± 0.030 | 0.072 ± 0.035 |
| NeuAc_2_Fuc_1_Hex_6_HexNAc_5_ | C | 1.228 ± 0.269 | 1.439 ± 0.297 |
| NeuAc_3_Hex_7_HexNAc_5_ | C | 0.036 ± 0.015 | 0.103 ± 0.042 |
| NeuAc_1_NeuGc_1_Fuc_1_Hex_6_HexNAc_5_ | C | 0.126 ± 0.036 | 0.202 ± 0.086 |
| NeuAc_1_Fuc_1_Hex_7_HexNAc_6_ | C | 0.588 ± 0.147 | 0.754 ± 0.145 |
| NeuAc_1_Hex_8_HexNAc_6_ | C | 0.075 ± 0.029 | 0.110 ± 0.045 |
| NeuGc_2_Hex_6_HexNAc_6_ | C | 0.014 ± 0.014 | 0.053 ± 0.031 |
| NeuAc_3_Hex_6_HexNAc_5_ | C | 0.657 ± 0.268 | 0.539 ± 0.162 |
| NeuAc_2_NeuGc_1_Hex_6_HexNAc_5_ | C | 0.045 ± 0.027 | 0.095 ± 0.036 |
| NeuAc_2_Hex_7_HexNAc_6_ | C | 0.077 ± 0.041 | 0.065 ± 0.033 |
| NeuAc_3_Fuc_1_Hex_6_HexNAc_5_ | C | 0.373 ± 0.114 | 0.459 ± 0.121 |
| NeuAc_2_NeuGc_1_Fuc_1_Hex_6_HexNAc_5_ | C | 0.036 ± 0.015 | 0.103 ± 0.095 |
| NeuAc_2_Fuc_1_Hex_7_HexNAc_6_ | C | 0.520 ± 0.176 | 0.665 ± 0.135 |
| NeuAc_1_NeuGc_1_Fuc_1_Hex_7_HexNAc_6_ | C | 0.038 ± 0.025 | 0.101 ± 0.041 |
| NeuAc_1_Fuc_1_Hex_8_HexNAc_7_ | C | 0.047 ± 0.030 | 0.059 ± 0.032 |
| NeuAc_4_Hex_6_HexNAc_5_ | C | 0.083 ± 0.043 | 0.085 ± 0.047 |
| NeuAc_3_Hex_7_HexNAc_6_ | C | 0.023 ± 0.014 | 0.016 ± 0.016 |
| NeuAc_2_Hex_8_HexNAc_7_ | C | 0.004 ± 0.004 | 0 |
| NeuAc_3_Fuc_1_Hex_7_HexNAc_6_ | C | 0.174 ± 0.077 | 0.206 ± 0.066 |
| NeuAc_2_Fuc_1_Hex_8_HexNAc_7_ | C | 0.033 ± 0.020 | 0 |
| NeuAc_1_Fuc_1_Hex_9_HexNAc_8_ | C | 0 | 0.043 ± 0.023 |
| NeuAc_4_Fuc_1_Hex_7_HexNAc_6_ | C | 0.046 ± 0.024 | 0.037 ± 0.023 |
| NeuAc_3_Fuc_1_Hex_8_HexNAc_7_ | C | 0.016 ± 0.010 | 0.016 ± 0.010 |
| NeuAc_2_Fuc_1_Hex_9_HexNAc_8_ | C | 0.008 ± 0.005 | 0 |
| NeuAc_4_Fuc_1_Hex_8_HexNAc_7_ | C | 0.002 ± 0.002 | 0 |
| NeuAc_3_Fuc_1_Hex_9_HexNAc_8_ | C | 0.001 ± 0.001 | 0 |
|  |  |  |  |
| Fuc_1_Hex_2_HexNAc_2_ | DP^c^ | 0.745 ± 0.190 | 0.997 ± 0.602 |
| Hex_3_HexNAc_2_ | DP | 0.891 ± 0.220 | 1.122 ± 0.308 |
| Fuc_1_Hex_3_HexNAc_2_ | DP | 2.402 ± 0.504 | 3.157 ± 0.943 |
| Hex_4_HexNAc_2_ | DP | 0.618 ± 0.106 | 0.755 ± 0.241 |
| Fuc_1_Hex_4_HexNAc_2_ | DP | 0.396 ± 0.095 | 0.069 ± 0.045 |

^a^ Glycans grouped as oligomannose (O), hybrid (H), complex (C) and degradation product (DP).

^b^ Percentage of total glycan signal ± SEM for n=5 are shown.

^c^ DP are small glycans that are not produced by the mammalian *N*-glycosylation machinery and therefore most likely originate from lysosomal degradation.
