# Supplementary figures and images for: Glycan Profiling Shows Unvaried N-Glycomes in MSC Clones with Distinct Differentiation Potentials
Source: Front Cell Dev Biol. 2016 May 31;4:52. doi: 10.3389/fcell.2016.00052 (PMC4885867; doi:10.3389/fcell.2016.00052)

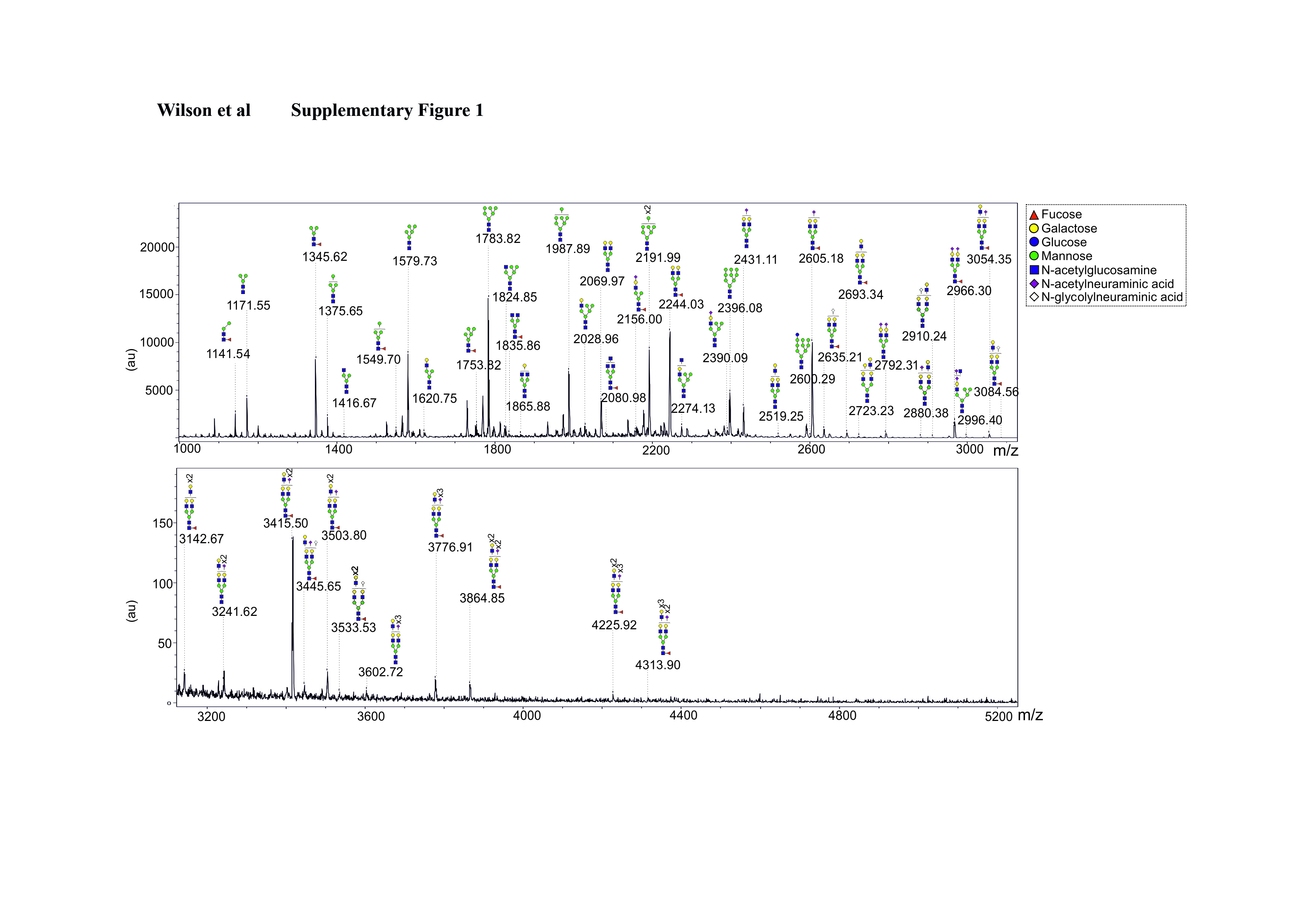

Supplement: Supplementary Figure 1 — Representative MALDI-TOF spectrum of permethylated N-glycans harvested from MSCs grown for 21 days in basal medium. For details see Figure 1. [file Image1.JPEG]

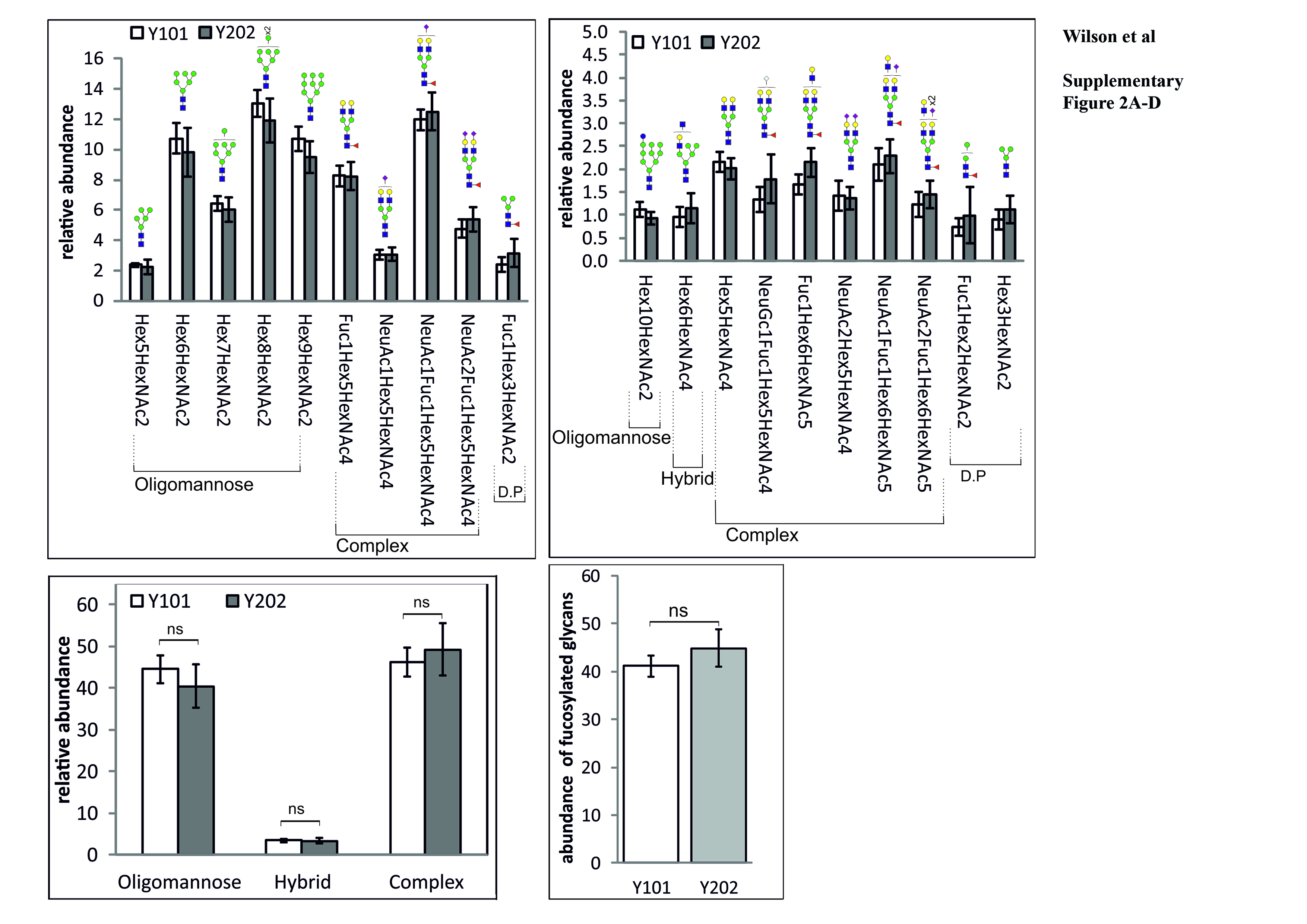

Supplement: Supplementary Figure 2 — Comparison of Y101 and Y202 N-glycans, for details see Figures 2C–F. Comparisons of averaged (n = 5) Y101 and Y202 normalized N-glycan total peak intensities of: (A) Individual glycan structures with abundances above 3% of the total. (B) Individual glycan structures with abundances of 1–3% of the total. D.P, degradation product, most likely produced in the lysosome. (C) Sums of different glycan types. (F) Sums of all fucosylated glycan abundances. Error bars show standard error of the mean. [file Image2.JPEG]

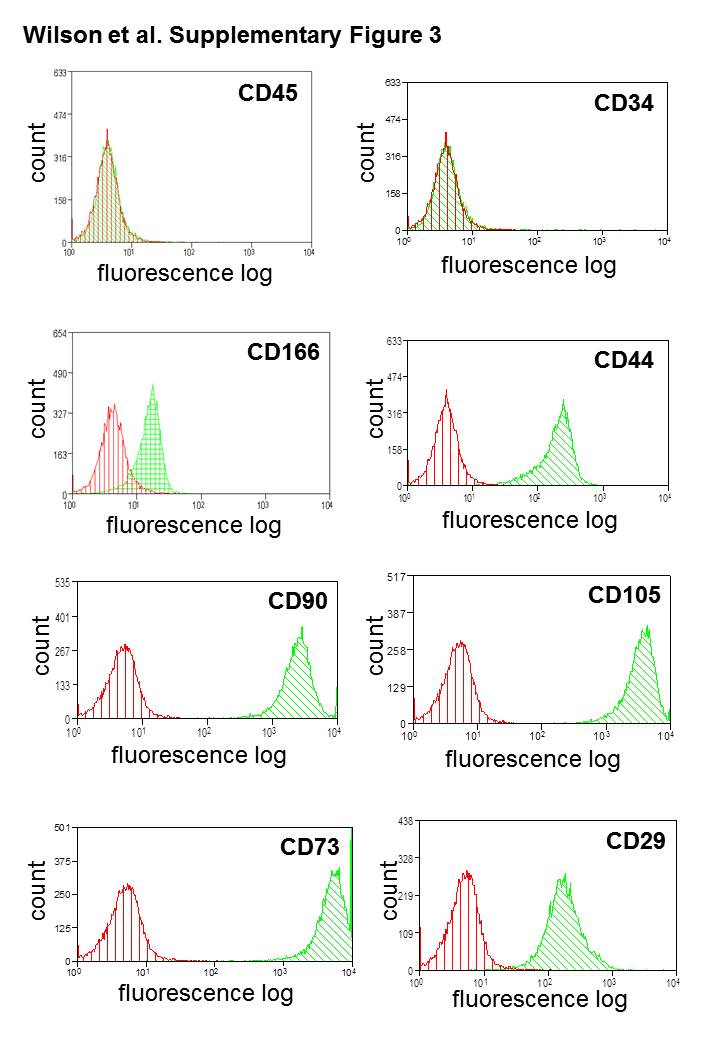

Supplement: Supplementary Figure 3 — Presence and absence of the indicated cell surface CD markers was determined for Y202 cells using flow cytometry. [file Image3.JPEG]

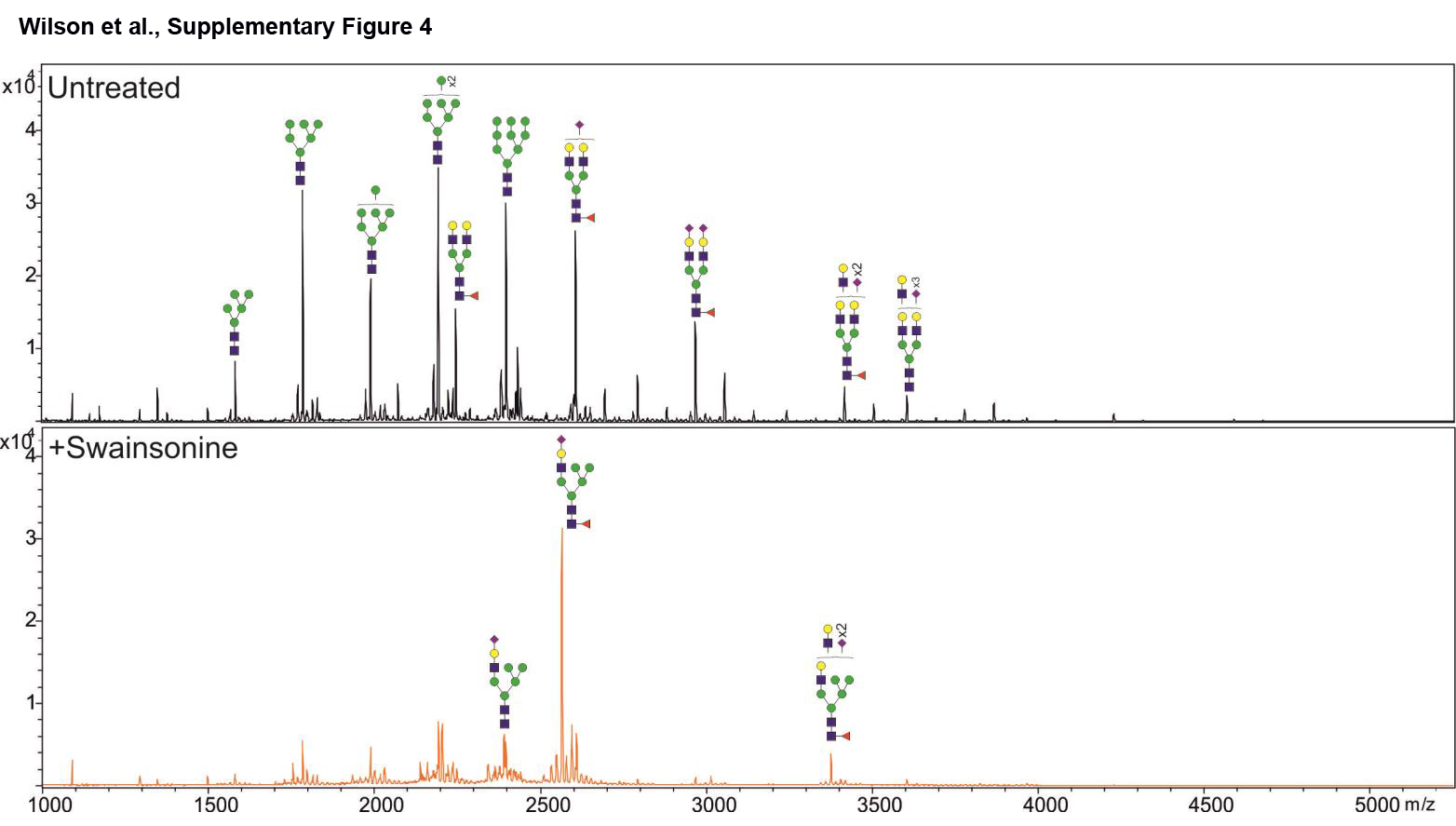

Supplement: Supplementary Figure 4 — Comparison of representative MALDI-TOF spectra of N-glycans harvested from Y101 MSCs cultured for 48 h in the presence or absence of swainsonine. The assigned glycan structures are only shown for the most prominent peaks. [file Image4.JPEG]
